# Drug-resistant menin variants retain high binding affinity and interactions with MLL1

**DOI:** 10.1016/j.jbc.2024.107777

**Published:** 2024-09-12

**Authors:** Joshua Ray, Bradley Clegg, Jolanta Grembecka, Tomasz Cierpicki

**Affiliations:** 1 Department of Pathology, University of Michigan, Ann Arbor, Michigan, USA; 2 Program in Chemical Biology, University of Michigan, Ann Arbor, Michigan, USA; 3 Department of Biophysics, University of Michigan, Ann Arbor, Michigan, USA

**Keywords:** protein–protein interaction, protein structure, mutant, drug resistance, ligand-binding protein, leukemia

## Abstract

Menin is an essential oncogenic cofactor of MLL1 fusion proteins in acute leukemias and inhibitors of the menin-MLL1 interaction are under evaluation in clinical trials. Recent studies found emerging resistance to menin inhibitor treatment in patients with leukemia as a result of somatic mutations in menin. To understand how patient mutations in menin affect the interaction with MLL1, we performed systematic characterization of the binding affinity of these menin mutants (T349M, M327I, G331R and G331D) and the N-terminal fragment of MLL1. We also determined the crystal structures of menin patient mutants and their complexes with MLL1-derived peptides. We found that drug-resistant mutations in menin occur at a site adjacent to the MLL1 binding site, but they do not affect MLL1 binding to menin. On the contrary, our structural analysis shows that all these point mutations in menin generate steric clash with menin inhibitors. We also found that mutation G331D results in a very slow dissociation of MLL1 from menin and this mutant might be particularly difficult to inhibit with small molecule drugs. This work provides structural information to support the development of a new generation of small molecule inhibitors that overcome resistance caused by menin mutations.

Menin, a 67 kDa protein encoded by the *MEN1* (*Multiple Endocrine Neoplasia 1*) gene, is a chromatin adaptor protein that interacts with MLL1 (Mixed Lineage Leukemia 1, also known as KMT2A) to regulate the expression of multiple genes, including *HOXA* cluster genes (1, 2, 3). Menin has been identified as an essential oncogenic co-factor of MLL1 fusion proteins found in acute leukemia patients (1, 3). The interaction between menin and MLL1 has been implicated in the development of acute leukemias driven by *MLL1/KMT2A* rearrangements (*MLL1*-r) (3, 4, 5, 6, 7), *NPM1* mutations (*NPM1*-mut) (8, 9, 10, 11, 12), and *NUP98* rearrangements (*NUP98*-r) (13, 14, 15). Indeed, the disruption of the menin–MLL1 interaction with small molecule inhibitors leads to pronounced anti-leukemic effects in pre-clinical models of leukemia with *MLL1-r* and *NPM1-mut* (11, 12, 14, 16, 17, 18). Optimized menin inhibitors, including Ziftomenib (19), Revumenib (20) and others, such as DS-1594, BMF-219, JNJ-75276617, and DSP-5336 have entered clinical trials (21). Results of the phase 1/2 first-in-human clinical trials testing Revumenib and Ziftomenib in patients with *MLL1*-r and *NPM1*-mut acute leukemias revealed promising outcomes with an over 30% rate of complete remission (19, 20). Importantly, recent clinical studies found emerging resistance to Revumenib treatment and patient relapse as a result of somatic mutations in *MEN1*, leading to point mutations of T349M, M327I/V, and G331R/D (22). Understanding how these mutations affect menin structure as well as the interaction with MLL1 and current menin inhibitors will be crucial in the development of a new generation of inhibitors maintaining their activity against menin mutants.

Our previous studies on the menin–MLL1 interaction found that menin binds a 46 amino acid fragment from the N-terminus of MLL1 with low nanomolar binding affinity (2). Two binding motifs on MLL1, MBM1, and MBM2 (menin binding motifs 1 and 2), are responsible for this strong interaction. MBM1 consists of amino acid residues 6 to 13 of MLL1 (K_d_ = 56 nM) and is separated by a poly-glycine linker from MBM2, which comprises residues 24 to 40 (K_d_ = 1 μM) (2, 23). MBM1 binds to the central cavity of menin, utilizing Phe9, Pro10, and Pro13 as energetic hotspots (2), which occupy well-defined hydrophobic pockets, and positively charged arginine residues (Arg8 and Arg12) that are involved in electrostatic interactions with the negatively charged binding site on menin (23). MBM2 also carries a strong positive charge due to multiple arginine residues, which maintain favorable electrostatic interactions with the central cavity on menin (23). The MBM1 binding site on menin has emerged as the primary binding site for inhibitors aiming to disrupt the interaction between menin and MLL1.

It was recently shown that mutations of M327I and T349M reduce the binding of Revumenib by 51- and 111-fold, respectively (22). The crystal structure revealed a direct clash between I327 and Revumenib. On the contrary, biochemical studies showed that M327I and T349M mutations in menin have a relatively minor impact on the affinity of MLL1 binding (22). However, systematic evaluation of how point mutations affect menin structure and interactions of all four mutants with MLL1 is currently missing. Computational simulations predicted that all these mutations result in structural perturbations in the menin binding site (22). To address this experimentally, we expressed the four most common menin variants found in AML patients and characterized their binding with MLL1 using biophysical and structural biology methods. We found that drug-resistant menin mutations do not impair the affinity of MLL1 binding. We also solved the crystal structures of these menin mutants, showing no structural changes in the MLL1 binding site on menin. Further structural analysis revealed that point mutations in menin occur in a site that is adjacent to the MLL1 MBM1 binding but has no direct contact or clash with MLL1. On the contrary, all these mutations sterically clash with the binding mode of the small molecule menin inhibitor Revumenib. We also discovered that the G331D mutant presents a particularly challenging target to inhibit, as this menin variant shows a very slow rate of dissociating from MLL1 as compared to the wild-type and other menin mutants. Overall, this work provides valuable structural information to support the development of a new generation of small molecule inhibitors that overcome resistance caused by menin mutations.

## Results

### Menin mutants maintain high-affinity interaction with MLL1

**Figure 1:**
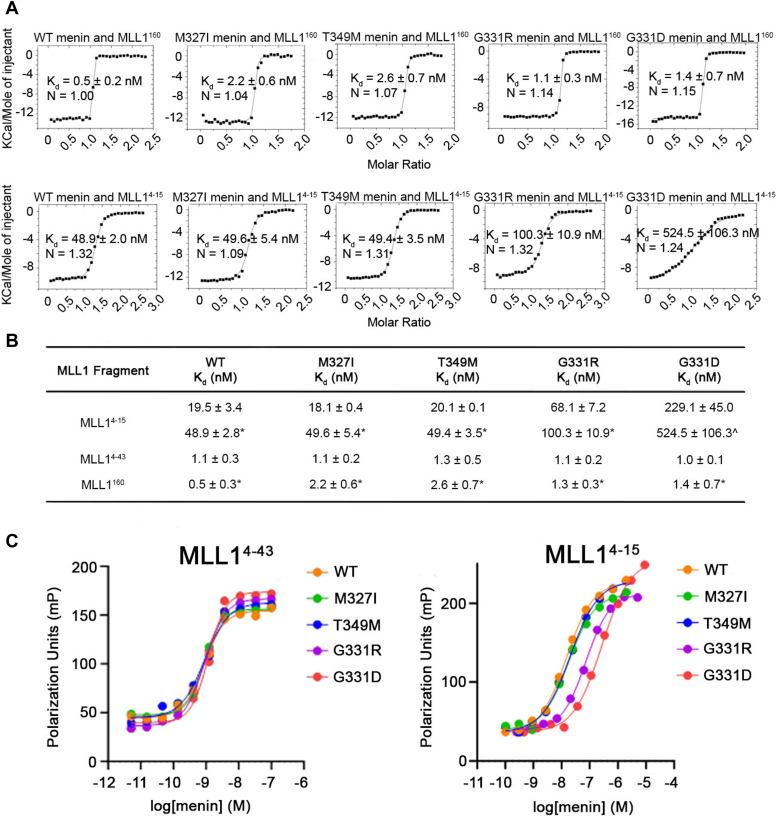
**Point mutations in menin have moderate effects on the binding affinity to MLL1**. *A*, representative binding isotherms from ITC experiments with menin^WT^ or menin mutants and MLL1^160^ or MLL1^4-15^. K_d_ values are provided, and N is stoichiometry. *B*, summary of K_d_ values for binding of menin mutants with MLL1^4-15^, MLL1^4-43^, and MLL1^160^ fragments determined using FP and ITC experiments. ∗ indicates K_d_ values measured from ITC experiments, n = 2 replicates, ˆ indicates n = 4 replicates. K_d_ obtained from FP experiments refers to the apparent K_d_ values. *C*, FP binding experiments for menin^WT^ and indicated mutants with FLSN-MLL1^4-43^ or FLSN-MLL1^4-15^.

Treatment of patients with AML with menin inhibitor Revumenib led to *MEN1* mutations conferring resistance to all advanced menin inhibitors (22). To understand how these mutations affect the interaction with MLL1, we tested the binding affinity of wild-type menin (menin^WT^) and four of the most common drug-resistant mutants, M327I (menin^M327I^), T349M (menin^T349M^), G331D (menin^G331D^), and G331R (menin^G331R^), with different MLL1 fragments. First, we confirmed that point mutations in menin do not disrupt menin stability using thermal shift assays (Fig. S1) (24). Then, we assessed the binding of MLL1 encompassing residues 1 to 160, containing the intact menin binding domain (MLL1^160^), by Isothermal Titration Calorimetry (ITC). We found that the binding affinity (K_d_) of MLL1^160^ to menin^WT^ and all four menin variants ranges between 0.5 to 2.6 nM, indicating that all these mutants maintain very strong binding to MLL1 (Fig. 1, *A* and *B*). Previous thermodynamic analysis of ITC data revealed that the menin-MLL1 interaction is driven by a favorable gain in enthalpy (2). Here, we found that all four menin variants also bind MLL1 with favorable enthalpy and show expected 1:1 stoichiometry (Fig. 1*A*, Table S1*A*).

The minimal fragment of MLL1 needed for the high-affinity interaction with menin is located within the first 43 amino acids of MLL1 and contains two menin binding motifs: MBM1 and MBM2 (2). Thus, we tested the binding of a fluorescein-labeled MLL1 fragment encompassing residues 4 to 43 (FLSN-MLL1^4-43^) to menin variants using the fluorescence polarization (FP) method (Fig. 1*C*). These experiments further confirmed that the binding affinity to MLL1 is not affected by menin patient mutations as the K_d_ values are very similar for these mutants, ranging from 1.0 to 1.3 nM (Fig. 1*B*). These results support that patient mutations in *MEN1* do not affect menin interactions with MLL1. MBM1 represents a high affinity menin binding motif spanning residues 6 to 13 in MLL1 (2). Structural studies revealed that MBM1 binds to a well-defined pocket on menin, which also represents the primary site for all reported small molecule menin inhibitors (23). The second, lower affinity binding motif, MBM2, contains multiple positively charged residues and further enhances the binding of MLL1 to menin (2), but the detailed interaction mode of MBM2 with menin is not known. To assess whether mutations in menin affect the binding of MBM1, we determined the K_d_ values using both ITC and FP. The affinities towards MLL1^4-15^ for menin^WT^ and two mutants, M327I and T349M, remained very similar, with K_d_ values ranging from 18 nM to 49 nM as assessed by FP and ITC (Fig. 1, *A* and *B*). Interestingly, we found that mutations of G331 to either arginine or aspartate decreased affinities towards MLL1^4-15^ by approximately 3- and 10-fold for G331R and G331D, respectively (Fig. 1*B*). However, reduced binding for G331 menin mutants was not observed for the longer MLL1^4-43^ and MLL1^160^ constructs (Fig. 1, *B* and *C*). This indicates that the presence of the second motif, MBM2, compensates for the partial loss of the MBM1 affinity towards G331 mutants.

### Menin mutants retain canonical binding to MBM1

**Figure 2:**
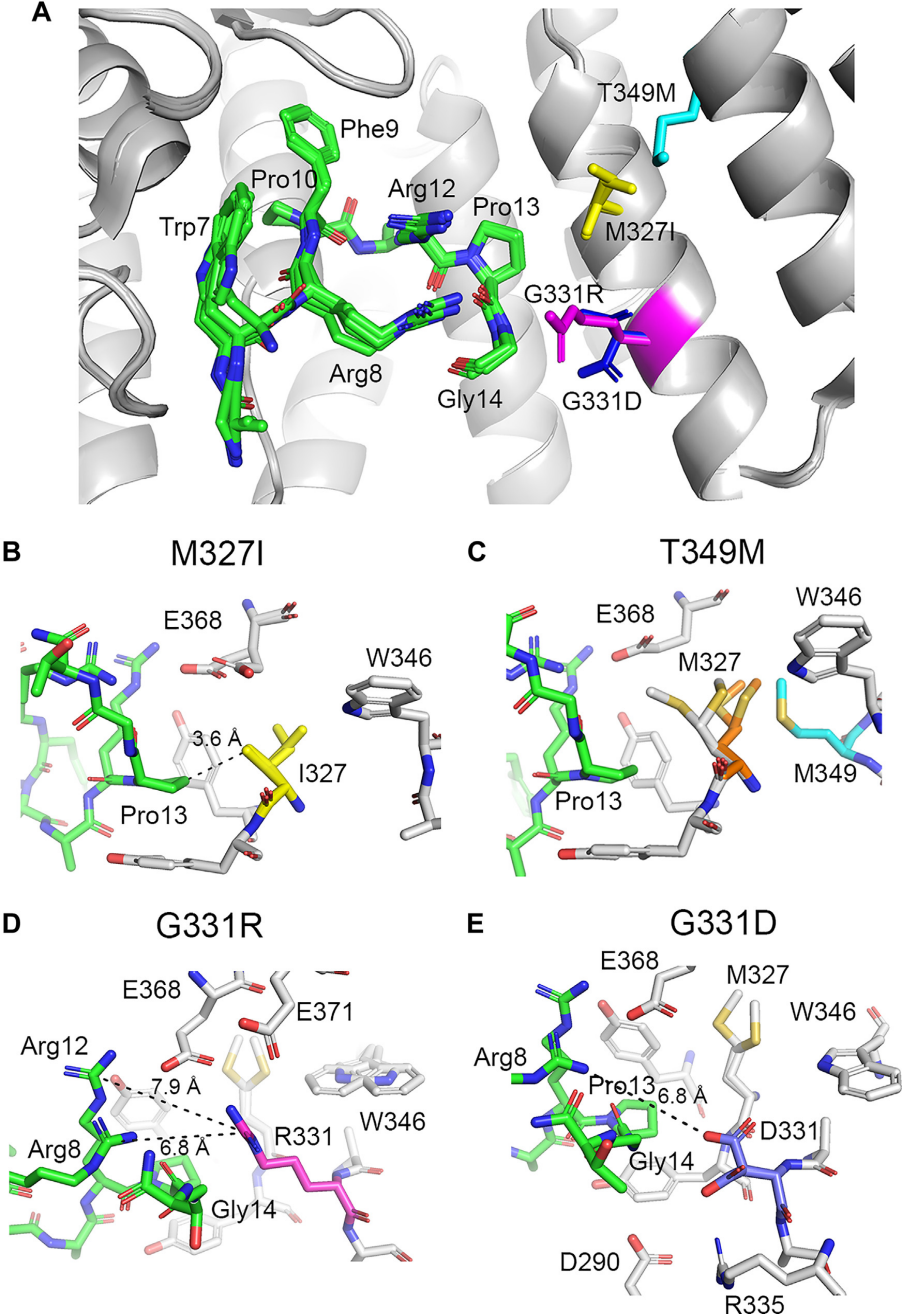
**Patient mutations in menin do not interfere with the binding of the MLL1 MBM1 motif**. *A*, superposition of the crystal structures of menin^WT^ and four mutants: menin^M327I^, menin^T349M^, menin^G331R^, menin^G331D^ (shown as *gray* ribbon) with bound MLL1^4-15^ (shown in sticks with *green* carbons). Mutated residues in menin are shown in distinct colors and labeled. Numbering of menin residues is according to the long menin isoform and includes +5 shift when referenced to the menin crystal structures deposited in PDB. *B*, details of the menin^M327I^-MLL1^4-15^ complex showing the distance between I327 (*yellow* carbons) and Pro13. *C*, details of the menin^T349M^-MLL1^4-15^ complex showing a conformational shift of M327 (*gray* carbons for menin^T349M^ and *orange* carbons for menin^WT^). Mutated M349 is shown with *cyan* carbons. *D* and *E*, crystal structures of menin^G331R^ and menin^G331D^ bound to MLL1^4-15^. Mutated residues R331 and D331 are shown with *magenta* and *blue* carbons, respectively.

To understand how menin mutations affect protein structure and interactions with MBM1, we determined high-resolution crystal structures of all four apo-menin mutants as well as their complexes with MLL1^4-15^. We found that point mutations in menin do not change the protein structure and only minimally perturb menin residues surrounding the MBM1 binding site (Fig. S2, Table S2). Subsequently, we determined the crystal structures of the four menin mutants in complex with MLL1^4-15^ (Table S3), and we found that all these menin variants bind MLL1^4-15^ in a nearly identical mode as wild-type menin (Fig. 2*A*). All inhibitor resistant mutations in menin are in a close vicinity to the MBM1 binding site near the Pro13 pocket but show no direct overlap with MLL1^4-15^ binding (Fig. 2*A*).

The M327I mutation replaces the side chain of methionine with the branched isoleucine comprising a C^γ2^ methyl located 3.6 Å from the Pro13 side chain of MBM1 (Fig. 2*B*), supporting no steric clash with the mutant. Furthermore, T349 is even more distant from Pro13, and the T349M mutation changes the conformation of M327, which adopts multiple conformations and approaches MBM1 as a result of the bulkier side chain of M349. However, similar to the M327I variant, the T349M mutant has no clash with Pro13 in MBM1 (Fig. 2*C*). Since both M327I and T349M mutations do not change the charge around the MBM1 binding site and do not introduce steric clashes, these mutations have no impact on the affinity of MBM1 to menin (Fig. 1*B*). On the contrary, mutations of G331 to arginine or aspartate perturb the charge in the MLL1 binding site on menin, resulting in decreased MBM1 binding affinity (Fig. 1*B*). The crystal structures of G331R and G331D revealed that both mutations do not show steric clashes with MBM1, and the side chains of R331 and D331 approach Gly14 from MBM1 (Fig. 2, *A*, *D*, and *E*). The relatively short, 7 to 8 Å, distance between the guanidine groups of R331 in the G331R variant and Arg8 and Arg12 in MBM1 likely results in unfavorable electrostatic interactions, which could be partially compensated by favorable contacts with E368 and E371 (Fig. 2*D*), leading to a modest, 3-fold reduction in the affinity to the G331R variant (Fig. 1*B*). Surprisingly, in the case of the G331D variant, introduction of a negatively charged D331 resulted in an even more pronounced (10-fold) decrease in the MBM1 binding affinity (Fig. 1*B*). The structure of menin^G331D^ with bound MBM1 shows that the side chain of D331 is ∼7 Å from the closest MBM1 arginine, Arg8, suggesting relatively modest favorable electrostatic interactions (Fig. 2*E*). However, analysis of the crystal structures reveals no clear rationale for such a loss in the MBM1 binding affinity to menin^G331D^. We have also calculated the electrostatic potential for the wild-type, G331R, and G331D menin variants and found that in all cases the MBM1 site on menin has a strong negative charge that is minimally perturbed by either of the two G331 mutations (Fig. S3). This analysis suggests that the electrostatic potential in menin is optimized to match the charge of the MBM1 motif in MLL1, and an introduction of either positive or negative charge distorts the charge complementarity between menin and MBM1, leading to a decrease in the binding affinity.

### Mutation G331D results in a very slow dissociation of MLL1 from menin

**Figure 3:**
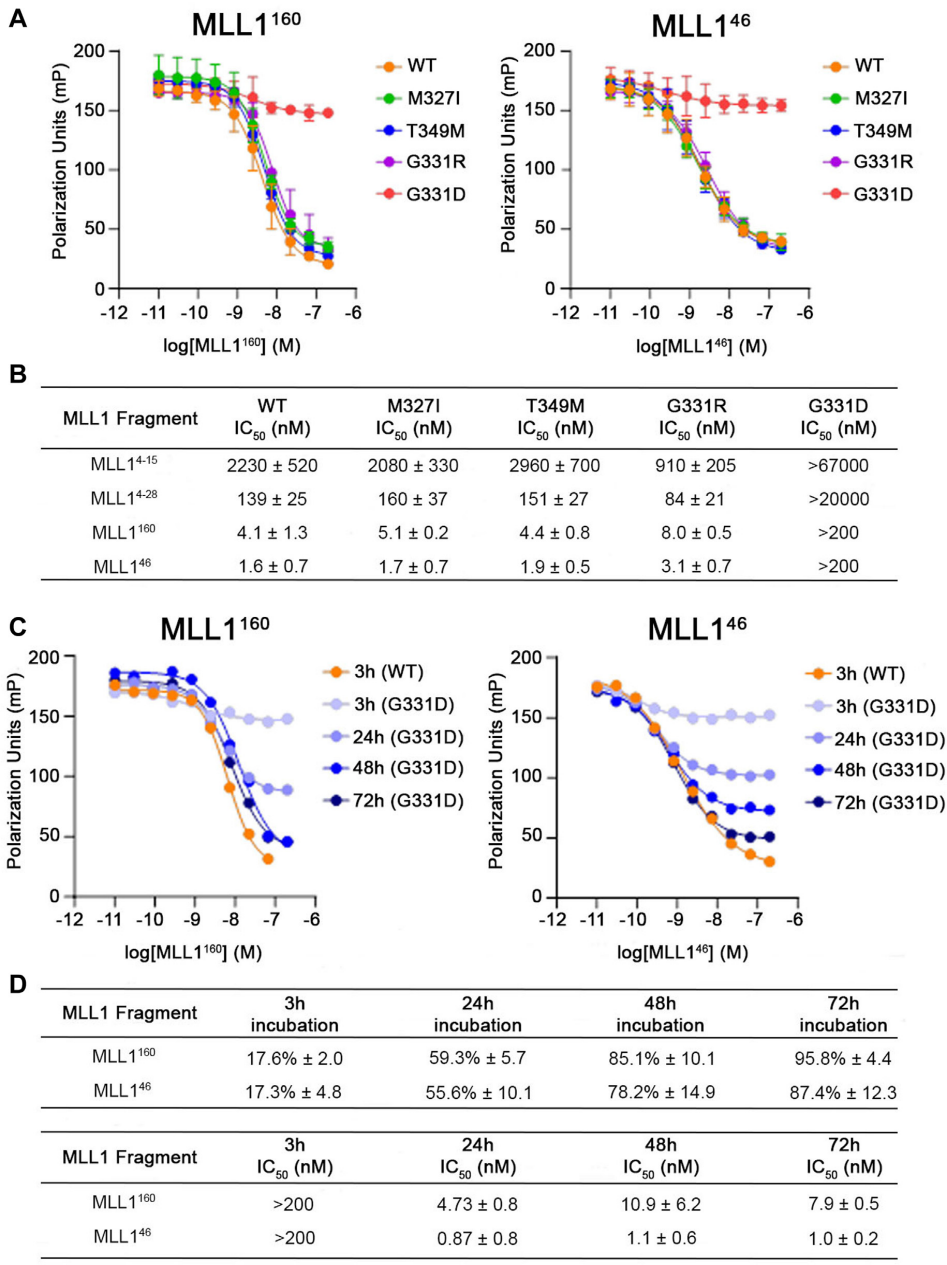
**The menin**^**G331D**^**-MLL1 complex shows slow dissociation when compared to other menin mutant complexes**. *A*, FP competition experiments showing the inhibition of menin variants bound to FLSN-MLL1^4-43^ by MLL1^160^ or MLL1^46^. *B*, IC_50_ values from FP competition experiments with MLL1^160^ and MLL1^46^ for all menin. N = 3 replicates. *C*, FP competition experiments showing time-dependent inhibition of menin^G331D^-FLSN-MLL1^4-43^ by MLL1^160^ or MLL1^46^. *D*, percent inhibition (calculated for 200 nM MLL1 fragments) and IC_50_ values determined from FP competition experiments for dissociation of menin^G331D^-FLSN-MLL1^4-43^ by MLL1^160^ or MLL1^46^ in a time-dependent manner. N = 3 replicates.

To better understand the mechanism of MLL1 binding with menin mutants, we performed FP competition experiments using FLSN-MLL1^4-43^ as a fluorescent probe for competitive displacement by MLL1 peptides (25). First, we tested competition with MLL1^160^ and found very similar IC_50_ values for wild-type menin and the three mutants M327I, T349M, and G331R, ranging between 4.1 and 8 nM (Fig. 3, *A* and *B*). Surprisingly, we found virtually no activity of MLL1^160^ with the menin^G331D^ mutant up to 200 nM, suggesting that this complex might be much more difficult to dissociate (Fig. 3*A*). Very similar results were observed when using MLL1^46^ (spanning amino acids 1–46 of MLL1, Fig. S4) as a competitor, with IC_50_ values between 1.7 and 3.1 nM for wild-type, M327I, T349M, and G331R menin (Fig. 3, *A* and *B*). Likewise, MLL1^46^ showed no activity with G331D up to 200 nM (Fig. 3*A*). Such an aberrant behavior of the G331D variant is unexpected, as the MLL1 binding data demonstrates very similar binding affinities when compared to the wild-type menin (Fig. 1*B*). We have hypothesized that the lack of competition with MLL1 for the menin^G331D^ mutant might result from a much slower dissociation rate of the menin^G331D^–MLL1 complex, and to evaluate this we performed time-dependent competition experiments with MLL1^160^ and MLL1^46^. Standard conditions in the FP assay include 3 h incubations of the menin-FLSN-MLL1^4-43^ complex with competitors. First, we tested whether longer incubation time affects inhibition of the menin^WT^-MLL1 complex and found consistent IC_50_ values over time for both, MLL1^160^ and MLL1^46^, indicating that the menin–MLL1 complex is stable over 72 h (Fig. S5). Then, we performed competition experiments with the G331D variant and found that incubations of 24 h or longer are needed to observe dissociation of the menin^G331D^–MLL1^4-43^ complex (Fig. 3*C*). This analysis shows consistent IC_50_ values at different time points, but longer incubations lead to more pronounced levels of inhibition to eventually achieve complete inhibition (Fig. 3, *C* and *D*). For example, IC_50_ values for the competition with MLL1^160^ range from 4.7 to 10.9 nM, but percent inhibition increased from 50% to 90% when comparing 24 to 72 h incubations. A similar effect was observed for the competition with MLL1^46^ (Fig. 3, *C* and *D*). These results demonstrate that menin^G331D^ binds MLL1 with a similar affinity as wild-type menin, but this complex undergoes a very slow dissociation and longer residence time, most likely due to the additional negative charge in the binding site introduced by the G331D mutation.

### Intact MBM2 is necessary for effective competition with menin-MLL1

MLL1 contains two menin-binding motifs (Fig. S4), and to understand the contribution from both motifs to the menin-MLL1 interaction, we compared the competition in blocking the interaction between menin-FLSN-MLL1^4-43^ with MLL1^46^ (containing MBM1 and MBM2) and MLL1^4-15^ (containing solely MBM1). While MLL1^46^ is a very potent, low nM competitor for all tested menin variants except for menin^G331D^, MLL1^4-15^ shows 3-orders of magnitude weaker activity (Figs 3, *B* and S6), demonstrating that loss of MBM2 substantially reduces the competition activity. Then, we tested a MLL1^4-28^ peptide containing MBM1 and part of MBM2 (Figs 3, *B* and S6), and found that including the extended MLL1 fragment increases the activity by 10 to 20 fold when compared to MLL1^4-15^ (Fig. 3*B*). However, no significant competition with MLL1^4-28^ was observed for the menin^G331D^ variant, even with increased incubation times (Fig. S6). Overall, these experiments demonstrate that an intact MBM2 plays a crucial role in competition involving MLL1 fragments and menin variants. However, MBM2 spans ∼17 amino acids and most likely occupies a distant site from MBM1 on menin, suggesting that development of menin inhibitors targeting both sites simultaneously is unlikely.

### Point mutations in menin reduce the activity of Revumenib due to steric clashes

**Figure 4:**
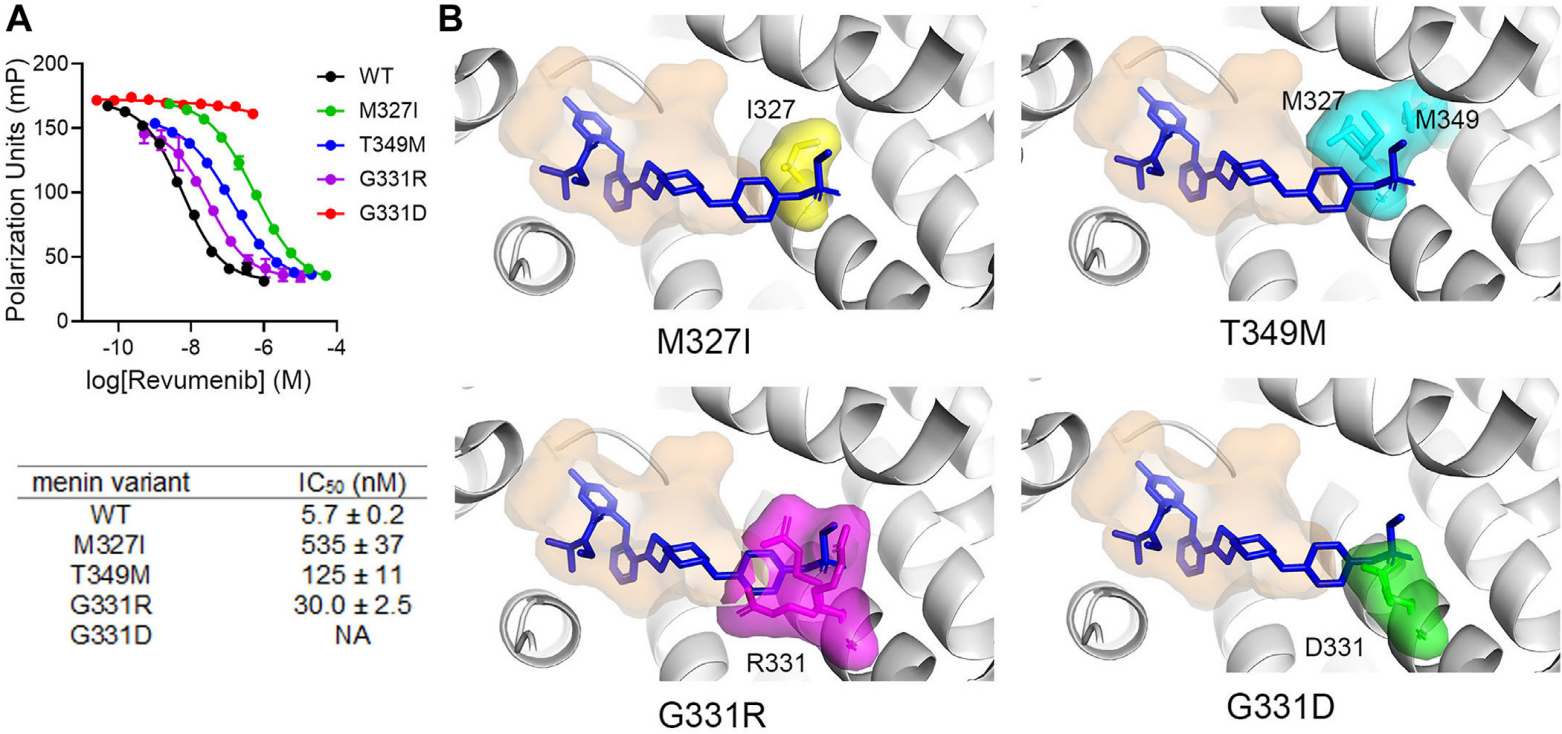
**Binding mode of Revumenib shows steric clashes with drug-resistant menin mutations**. *A*, FP competition experiments showing the inhibition of menin variants bound to FLSN-MLL1^4-43^ by Revumenib. Table shows IC_50_ values ± standard deviations, N = 3 replicates. NA – no activity. *B*, superposition of the crystal structures of menin mutants with the structure of the menin-Revumenib complex (PDB code 7UJ4). Revumenib is shown in blue sticks, mutated menin residues are in sticks with semi-transparent surfaces, distinct colors, and are labeled. The space occupied by MLL1^4-15^ is shown as an orange surface.

To understand the impact of menin mutations on the activity of Revumenib, we tested inhibition of the menin-FLSN-MLL1^4-43^ complex and found that the inhibitor is very active against wild-type protein (IC_50_ = 5.7 nM). However, activity against menin mutants is significantly reduced, from 5-fold for G331R to over 100-fold for M327I (Fig. 4*A*). As expected, no activity is observed for the G331D variant due to slow dissociation of the menin^G331D^–MLL1 complex. To evaluate the effect of mutations on inhibitor binding, we overlapped the crystal structures of menin mutants onto the structure of menin in complex with Revumenib. Biochemical studies have shown that the activity of Revumenib and other advanced menin inhibitors are strongly impaired by these mutations, with over 1 to 2 orders of magnitude loss in potency (22). Such an effect is observed for even the least perturbing mutations, such as M327I and T349M. Our structural analysis clearly shows that all these point mutations exhibit a steric clash with the menin inhibitor Revumenib, overlapping with inhibitor fragments that extend beyond the Pro13 binding pocket on menin (Fig. 4*B*). This example demonstrates that menin mutations resulting in even modest perturbations of the MLL1 binding site induce strong resistance to the small molecule menin inhibitors without affecting the interaction with the endogenous protein partner.

## Discussion

Menin is an attractive target for developing small-molecule inhibitors as novel drugs for acute leukemia (21, 26, 27, 28). Results from initial Phase 1/2 clinical studies revealed promising efficacy of menin inhibitors (*e.g.* Revumenib and Ziftomenib) in AML patients with *KMT2A* (*MLL1*) rearrangements and *NPM1* mutations, with ∼45% overall response rate and ∼35% complete remission found for both drugs (29). Nevertheless, the emergence of drug-resistant mutations calls for a need to develop a new generation of menin inhibitors, and it raises the question of how such mutations impact the menin–MLL1 interaction. Recent studies found that one of the most frequent mutations, M327I, impairs the activity of Revumenib through a direct steric clash between I327 and the inhibitor (22). Interestingly, the M327I mutation has no effect on the affinity of MLL1 binding (22). However, the effect of other patient mutations on menin structure and interactions with MLL1 have not been systematically investigated. Here, we demonstrated that the four most common drug resistant menin variants identified in patients with AML retain a high binding affinity to MLL1. In contrast to the predictions from computational studies (22), the crystal structures of the menin mutants revealed that these point mutations have no impact on menin structure or stability. Additionally, despite the close proximity of all point mutations to the MLL1 binding site on menin, they have no direct overlap with the high affinity menin binding motif (MBM1) of MLL1. Furthermore, the crystal structures of menin complexes with MLL1^4-15^ demonstrated the same binding mode of MBM1 with all menin variants. A series of competition experiments revealed that three menin mutants, M327I, T349M, and G331R, interact with MLL1 similarly as with the wild-type menin, but G331D has a strikingly different behavior. Point mutation G331D reduces the binding of the high-affinity MBM1 motif of MLL1 by 10-fold yet maintains the potent interaction with longer MLL1 fragments and shows a very slow rate of dissociation of MLL1. This demonstrates the complex impact of electrostatics on the potency and kinetics of protein-protein interactions. Additionally, an intact MBM2 motif is necessary for effective competition for binding of MLL1 to menin variants, indicating that patient mutations have no major impact on MBM2 binding.

The activity of Revumenib is significantly reduced by point mutations in menin as validated in our studies. The occurrence of these mutations sparks a new challenge for the development of menin-MLL1 inhibitors that efficiently bind to both wild-type menin and menin patient mutations. Our studies provide valuable structural data that may support the development of a new generation of menin inhibitors to avoid steric clashes with the mutant residues in the MLL1 binding site. While MBM2 is important for the high-affinity menin–MLL1 interaction, this second motif most likely binds in a distant site from the druggable MBM1 pocket on menin and thus cannot be engaged in the design of small molecule inhibitors. The unexpected kinetic effect of the G331D mutation and the slow dissociation of menin^G331D^-MLL1 suggests that this menin variant will be particularly difficult to inhibit with small molecule inhibitors and emphasizes the complexity of protein-protein interactions. Our studies support that the new generation of menin inhibitors should be designed to more closely mimic the interactions utilized by MBM1 rather than extend beyond this site to limit the resistance to *MEN1* mutations.

## Experimental procedures

### Cloning, expression, and Purification

The gene encoding full-length human menin was cloned into a pET32a expression vector (Novagen), and the generation of mutant menin constructs was performed using the QuikChange Site-Directed Mutagenesis Kit protocol (2, 16). Full-length human menin was expressed in BL21(DE3) cells (Invitrogen) and purified using a HisTrap FF (Cytiva) affinity chromatography column followed by thioredoxin-His_6_ tag cleavage using thrombin protease. The tag and menin were then separated by reverse-nickel using a HisTrap FF column, and any remaining contaminants were removed by anion exchange with a Q Sepharose HP column (GE Healthcare). In the final step, menin was dialyzed to a 50 mM Tris pH 7.5, 50 mM NaCl, 1 mM TCEP buffer and frozen at −80 °C for further experiments (2).

For crystallography experiments, a truncated version of menin with deletions of 3 internal fragments and the C-terminus was cloned into a pET32a expression vector (Novagen), and the generation of mutant menin crystallography constructs was performed using the QuikChange Site-Directed Mutagenesis Kit protocol (23).

Crystallography menin was expressed and purified as described for full-length menin, using TEV protease instead of thrombin. Additionally, after anion exchange, the crystallography protein was subjected to size exclusion chromatography using a HiLoad 16/60 Superdex 75 pg column (GE Healthcare). The protein was frozen at −80 °C in a 50 mM Tris pH 8.0, 50 mM NaCl, 1 mM TCEP buffer at 2.8 mg/ml for further experiments (23).

The gene encoding the N-terminal 160 amino acids of human MLL1 (MLL1^160^) was also cloned into a pET32a expression vector (Novagen). To avoid oxidation and dimerization of MLL1^160^, a single cysteine residue in the N terminus (Cys^2^) was mutated to an alanine (2).

MLL1^160^ was expressed in BL21(DE3) cells and collected as an insoluble fraction. The inclusion bodies were purified from the cell pellet and solubilized in a 6 M guanidine hydrochloride solution. On-column refolding was done using a HisTrap FF column and a 50 mM Tris pH 7.5, 200 mM NaCl, 40 mM Imidazole, 1 mM βME wash. The thioredoxin-His_6_ tag was then cleaved using precision protease and separated from MLL1^160^ using cation exchange with an SP Sepharose HP column (GE Healthcare). In the final step, MLL1^160^ was dialyzed to a 50 mM Tris pH 7.5, 150 mM NaCl, 1 mM TCEP buffer, and frozen at −80 °C for further experiments (2).

To obtain a shorter fragment of MLL1 with only the menin binding domain (MBD), a stop codon mutation was introduced after amino acid 46, generating MLL1^46^. MLL1^46^ was expressed as a soluble protein and was purified using a HisTrap FF affinity chromatography column followed by thioredoxin-His_6_ tag cleavage with precision protease. Cation exchange with an SP Sepharose HP column separated the tag and protein, and MLL1^46^ was dialyzed to a 50 mM Tris pH 7.5, 150 mM NaCl, 1 mM TCEP buffer and frozen at −80 °C for further experiments (2).

### Peptides

All peptides (>95% purity) were ordered from GenScript. FLSN-MLL1^4-43^ contains residues SARWRFPARPGTTGGGGGGGRRGLGGAPRQRVPALLLPPG with the N-terminal fluorescein and C-terminal amide.

### Thermal stability experiments

Thermal stability experiments to measure the melting temperature (T_M_) of menin variants were performed using the Bio-Rad CFX96 Real-time System, C1000 Touch Thermal Cycler. Samples of 5 μM menin were prepared in a 50 mM Tris pH 7.5, 50 mM NaCl, 1 mM TCEP, 5x SYPRO Orange solution, and incubated for 1 h. Samples were then distributed into a PCR plate in quadruplets at 25 μl volumes and subjected to a temperature gradient from 10 to 105 °C with fluorescence scanning. T_M_ was determined using Bio-Rad CFX Manager software, and figures were prepared using GraphPad Prism 10. All thermal stability experiments were run in triplicate.

### Isothermal Titration Calorimetry (ITC)

Menin and MLL1 fragments were dialyzed at 4 °C against a 50 mM sodium phosphate pH 7.5, 50 mM NaCl, 1 mM TCEP buffer, and degassed prior to measurement. The titrations were performed using the VP-ITC system (MicroCal) at 25 °C. The calorimetric cell, which contained menin (concentrations in the 5–10 μM range), was titrated with the MLL1^160^ and MLL1^4-15^ derived peptides (concentration range 40–100 μM) injected in 10-μL aliquots. Data was analyzed using Origin 7.0 (OriginLab) to obtain K_d_, stoichiometry, entropy, and enthalpy (2). Experiments with WT, M327I, T349M, and G331R menin and MLL1^160^ or MLL1^4-15^ were run in duplicate. Experiments with G331D menin and MLL1^160^ were run in duplicate while experiments with G331D and MLL1^4-15^ were run in quadruplicate.

### Fluorescence polarization (FP) experiments

FP binding experiments utilized MLL1-derived peptides, FLSN-MLL1^4-43^ at 4 nM and FLSN-MLL1^4-15^ at 10 nM, titrated against a range of menin concentrations in the FP buffer (50 mM Tris pH 7.5, 50 mM NaCl, 1 mM TCEP) to obtain K_d_ measurements. Protein-peptide complexes were incubated for 1 h before measuring the change in fluorescence polarization and anisotropy at 525 nm after excitations at 495 nm using the PHERAstar microplate reader (BMG) (2).

FP competition experiments used FLSN-MLL1^4-43^ at 4 nM and menin at 4 nM against varying concentrations of unlabeled MLL1 peptides in FP buffer for IC_50_ determination. Protein-peptide complexes were incubated for 1 h before unlabeled peptide competitors were added to ensure complete binding. Unlabeled peptide competitors were incubated with the protein-peptide complexes for 3 h before measurement using the PHERAstar microplate reader (25). Experiments with Revumenib were run with the same steps and conditions but used 5% DMSO in the final solution. K_d_ and IC_50_ values were calculated using GraphPad Prism 10 by plotting mP values measured for each protein/MLL1 peptide or Revumenib as a function of protein/MLL1 peptide (or Revumenib) concentration (25). All FP binding and competition experiments were run in triplicate.

### Crystallization of menin and menin complexes

For crystallization experiments, 2.8 mg/ml of menin alone or incubated with MLL1^4-15^ peptide (GenScript) in a 1:4 M ratio were used. Crystals were obtained using the sitting-drop technique at 12 °C in 0.2 M lithium sulfate monohydrate, 0.1 M HEPES pH 7.5, and 25% (w/v) PEG-3350. During harvesting, crystals were protected using a cryosolution containing crystallography conditions, 20% PEG-550 MME, and 0.114 mM MLL1^4-15^ peptide (when present) followed by flash-freezing in liquid nitrogen (23).

### Crystallographic data collection and structure determination

Diffraction data for menin^M327I^, menin^T349M^, menin^G331D^ and menin^M327I^-MLL1^4-15^, menin^T349M^-MLL1^4-15^, menin^G331D^-MLL1^4-15^ cocrystal complexes were collected on beamline 21-ID-G at the Life Sciences Collaborative Access Team (Advanced Photon Source, Argonne National Laboratory) at a 0.97857 Å wavelength under liquid nitrogen streaming. The collected data was processed with the HKL2000 v722 package (30). Diffraction data for menin^G331R^ and menin^G331R^-MLL1^4-15^ cocrystal complex was collected at SOLEIL Beamline PROXIMA-2 at a 0.9801 Å wavelength under liquid nitrogen streaming. The collected data was processed with the XDS version Jun 30, 2023 package and AIMLESS v0.7.15 package. Structures of menin^M327I^, menin^T349M^, menin^G331R^, and menin^G331D^ were determined by molecular replacement using MOLREP with the structure of human menin^WT^ (PDB code: 4GPQ) as a search model. Structures of menin-MLL1^4-15^ complexes for menin^M327I^- MLL1^4-15^, menin^T349M^- MLL1^4-15^, menin^G331R^- MLL1^4-15^, and menin^G331D^- MLL1^4-15^ were determined by molecular replacement using MOLREP with the menin^WT^-MLL1^4-15^ cocrystal structure (PDB code: 4GQ6) as a search model. The models were refined using REFMAC (31), COOT (32), CCP4 (33), and PHENIX (34), packages.

## Data availability

The crystal structure of menin^WT^ (PBD code: 4GPQ) was used as the search model to determine the apo structures reported in this study. The structures of menin^M327I^, menin^T349M^, menin^G331R^, and menin^G331D^ have been deposited in the PDB under the accession codes 9C4X, 9C4Y, 9C4W, 9C4Z, respectively. The crystal structure of menin^WT^-MLL1^4-15^ (PDB code: 4GQ6) was used as the search model to determine the cocrystal structures reported in this study. The cocrystal structures of menin^M327I^, menin^T349M^, menin^G331R^, and menin^G331D^ with MLL1^4-15^ have been deposited in the PDB under the accession codes 9C4T, 9C4U, 9C4S, 9C4V, respectively. All main text data are in this manuscript.

## Supporting information

This article contains supporting information.

## Conflict of interest

The authors declare the following financial interests/personal relationships which may be considered as potential competing interests:

Drs. Grembecka and Cierpicki received research support from Kura Oncology, Inc. They have also served as consultants for Kura Oncology, have equity ownership in the company and are co-inventors on patent applications covering menin inhibitors. Drs. Grembecka and Cierpicki receive royalties from the University of Michigan on the patents covering menin inhibitors that were licensed to Kura Oncology.
