# Supplementary material for: Drug-resistant menin variants retain high binding affinity and interactions with MLL1
Source: J Biol Chem. 2024 Sep 12;300(10):107777. doi: 10.1016/j.jbc.2024.107777 (PMC11490872; doi:10.1016/j.jbc.2024.107777)
Supplement: Supplemental Figures S1–S5 and Tables S1–S3 [file mmc1.pdf]

Title

Drug-resistant menin variants retain high binding affinity and interactions with MLL1

Authors

Joshua Ray[1]*, Bradley Clegg[1,2]*, Jolanta Grembecka[1,2,#] and Tomasz Cierpicki[1,2,3,#]

[1] Department of Pathology, University of Michigan, Ann Arbor, MI, USA

[2] Program in Chemical Biology, University of Michigan, Ann Arbor, MI, USA

[3] Department of Biophysics, University of Michigan, Ann Arbor, MI, USA

*Equal contribution

[#]Corresponding authors: jolantag@umich.edu; tomaszc@umich.edu

Supplementary data

Supplementary Figures

A

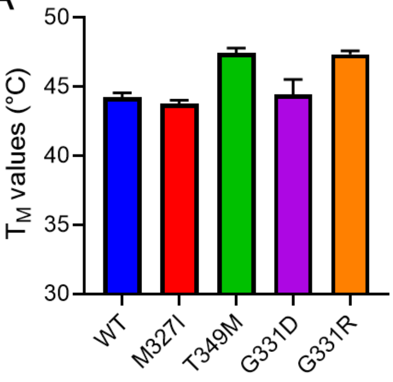

B

Menin variants	T_M values (°C)	ΔT_M relative to wild-type menin (°C)
WT	44.2 ± 0.3	0
M327I	43.8 ± 0.2	-0.5 ± 0.2
T349M	47.5 ± 0.3	3.2 ± 0.2
G331R	47.3 ± 0.2	3.1 ± 0.3
G331D	44.4 ± 1.3	0.2 ± 0.9

Supplementary Figure 1. Stability of menin variants determined using thermal shift assay. A. Comparison of thermal stability values (T_M) for menin variants. **B**. Table with T_M and ΔT_M values. N = 3.

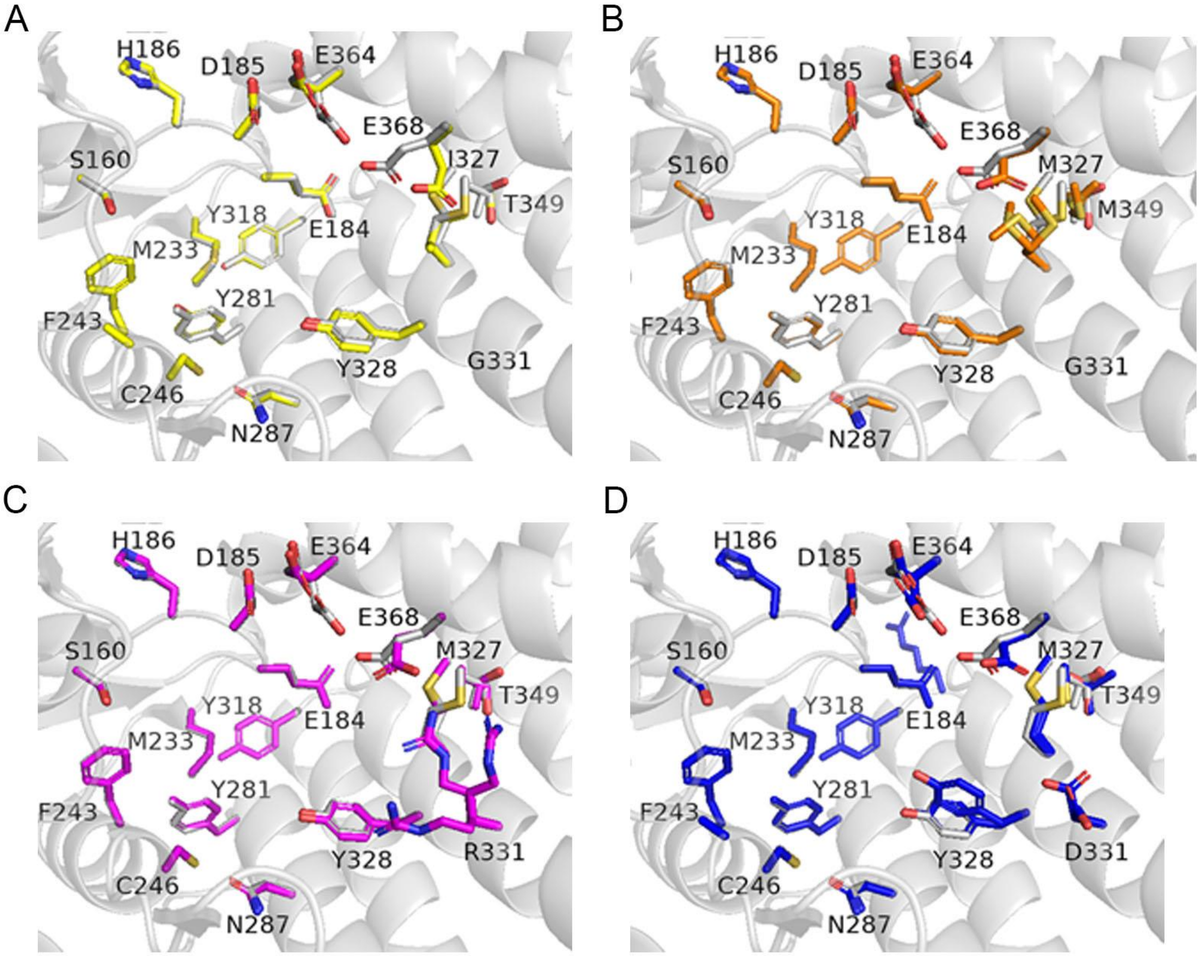

Supplementary Figure 2. **Crystal structure of menin mutants showing MBM1 binding site.** Panels show superposition of the crystal structure of menin^{WT} (PDB code 4GPQ; shown as gray ribbon, selected side chains shown as sticks with gray carbons) with structures of menin mutants: **A.** menin^{M327I} (PDB code 9C4X; carbon atoms in yellow), **B.** menin^{T349M} (PDB code 9C4Y; carbon atoms in orange), **C.** menin^{G331R} (PDB code 9C4W; carbon atoms in magenta) and **D.** menin^{G331D} (PDB code 9C4Z; carbon atoms in blue). Numbering of menin residues is according to the long menin isoform and include a +5 shift when referenced to the menin crystal structures deposited in the PDB.

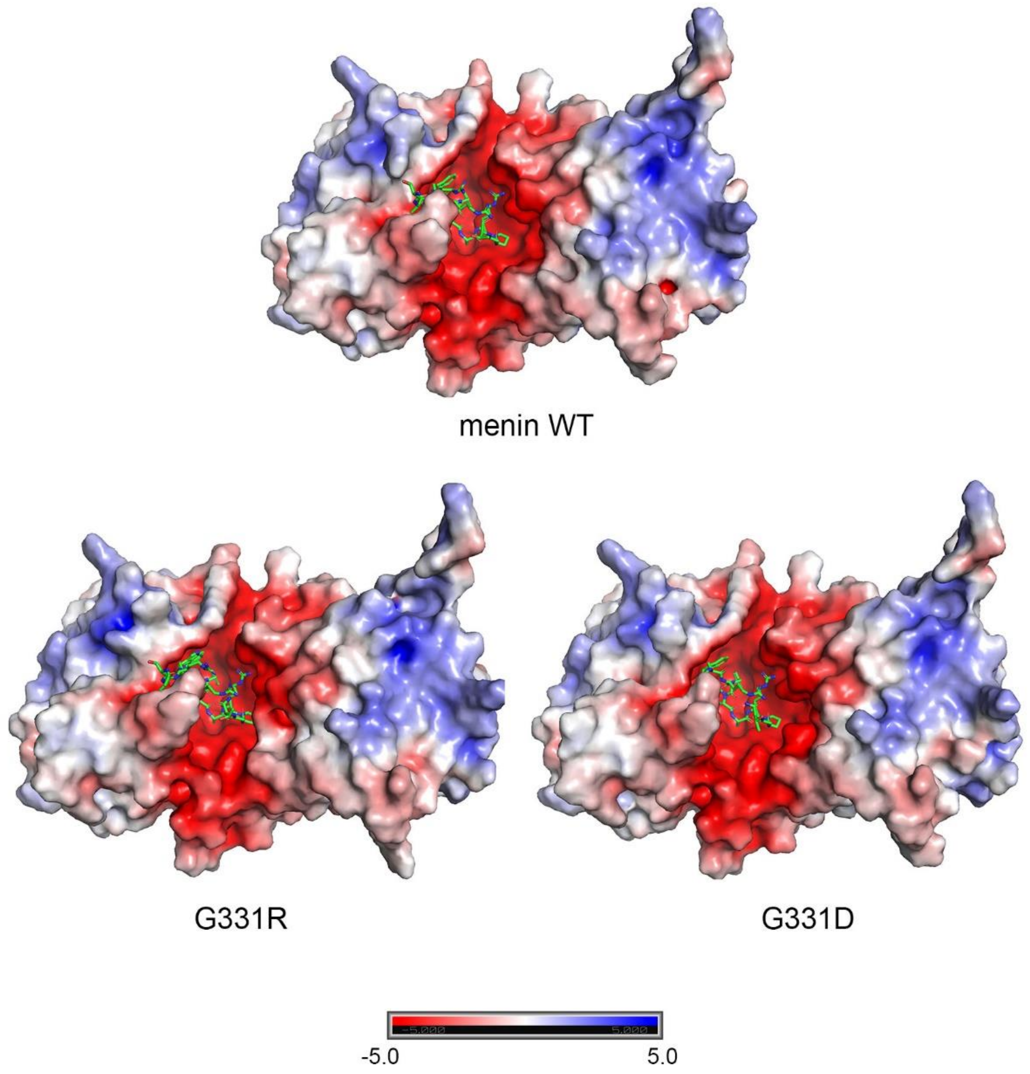

Supplementary Figure 3. Electrostatic potential calculated for menin^{WT}-MLL1$^{4\text{-}15}$, menin^{G331R}-MLL1$^{4\text{-}15}$ and menin^{G331D}-MLL$^{4\text{-}15}$ and mapped onto surface of menin, calculated using APBS server {Jurrus E, 2018 #53}. Scale is in dimensionless units $(k_b \times T)/e_c$, where k_b is the Boltzmann constant, $1.3806504 \times 10^{-23}$ J × K^{-1}, T is the temperature in K, and e_c is the charge of an electron, $1.60217646 \times 10^{-19}$ C.

$MLL1^{46}$	MAHSARWRFPARPGTTGGGGGGGRRGLGGAPRQRVPALLLPPGPPV	MBM1 (RWRFPARP); poly-glycine; MBM2 (RRGLGGAPRQRVPALLL)
$MLL1^{4-15}$	SARWRFPARPGT	MBM1 (RWRFPARP)
$MLL1^{4-28}$	SARWRFPARPGTTGGGGGGGRRGLG	MBM1 (RWRFPARP); poly-glycine

Supplementary Figure 4. Sequences of MLL1 fragments used in competition experiments showing residues in MBM1 and MBM2 motifs.

A

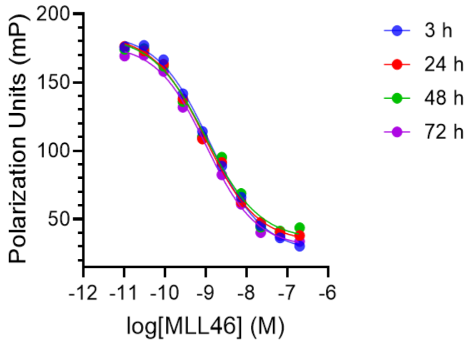

	3 h	24 h	48 h	72 h
IC50	1.258e-009	9.691e-010	1.018e-009	1.022e-009

B

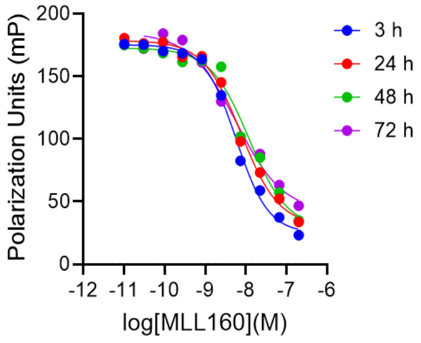

	3 h	24 h	48 h	72 h
IC50	5.768e-009	7.611e-009	1.196e-008	5.852e-009

C

WT Data												
Peptide	Parallel or Perpendicular	Time point	Absolute fluorescence values at decreasing (--->) peptide concentrations									
MLL1^{46}	Concentration (M) -->		2E-07	6.67E-08	2.22E-08	7.41E-09	2.47E-09	8.23E-10	2.74E-10	9.14E-11	3.05E-11	1.02E-11
	Parallel	3 hours	30299	29807	30829	32486	34762	37982	41998	44861	46005	46277
		72 hours	27959	28942	30069	32267	34975	38082	39551	43011	42145	41564
	Perpendicular	3 hours	28513	27714	28156	28468	29092	30203	31575	32053	32176	32460
		72 hours	26135	26826	27743	28555	29644	30571	30337	31271	29913	29534
MLL1^{160}	Concentration (M) -->		2E-07	6.67E-08	2.22E-08	7.41E-09	2.47E-09	8.23E-10	2.74E-10	9.14E-11	3.05E-11	1.02E-11
	Parallel	3 hours	40426	41753	44081	46431	55727	62520	65629	64989	64041	62523
		72 hours	53264	59638	72150	73911	91215	112312	104604	101214	95836	94933
	Perpendicular	3 hours	38595	38741	39181	39353	42477	44928	46700	46086	44928	43825
		72 hours	48499	52539	60468	60271	70223	81125	72823	69713	67675	64230

Supplementary Figure 5. Time dependent inhibition FP competition experiments showing the inhibition of menin^{WT}-MLL1 by MLL1^{46} (A) and MLL1^{160} (B). C. Comparison of the raw fluorescence signal between 3 and 72 h incubations from the FP competition experiments.

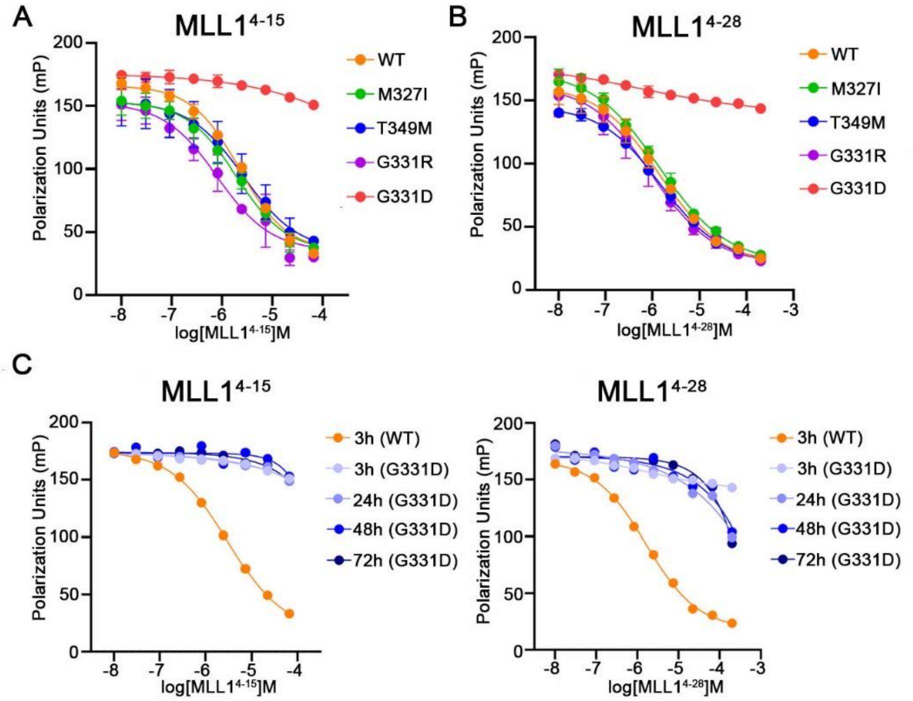

MLL1 Fragment	3h incubation	24h incubation	48h incubation	72h incubation
MLL1$^{4-15}$	17.0% ± 0.6	16.9% ± 2.3	16.9% ± 2.3	17.6% ± 0.2
MLL1$^{4-28}$	20.1% ± 0.9	57.3% ± 3.4	57.9% ± 15.3	55.5% ± 11.9

D

G331D Data

Peptide	Parallel or Perpendicular	Time point	Absolute fluorescence values at decreasing (--->) peptide concentrations									
MLL1^{46}	Concentration (M) -->		2E-07	6.67E-08	2.22E-08	7.41E-09	2.47E-09	8.23E-10	2.74E-10	9.14E-11	3.05E-11	1.02E-11
	Parallel	3 hours	39695	46622	44252	48140	47503	45637	47231	48171	49475	50432
		72 hours	24630	29453	30342	33622	35418	36038	41350	44175	45003	44218
	Perpendicular	3 hours	29198	34457	32543	35667	35150	33436	34369	34454	34934	35276
		72 hours	22234	26618	27290	29329	30016	28730	31267	32068	34968	31262
MLL1^{160}	Concentration (M) -->		2E-07	6.67E-08	2.22E-08	7.41E-09	2.47E-09	8.23E-10	2.74E-10	9.14E-11	3.05E-11	1.02E-11
	Parallel	3 hours	56705	56809	56853	57781	57689	59719	59994	61007	60951	59321
		72 hours	52740	55287	66561	75582	89923	93626	102115	88661	101292	95367
	Perpendicular	3 hours	42096	42400	42225	42429	42617	43054	43295	43428	43265	42113
		72 hours	48141	50092	56941	60376	66536	66786	71350	62445	70108	66252

Supplementary Figure 6. Competition experiments showing the inhibition of menin variants bound to FLSN-MLL1$^{4-43}$ by different MLL1 fragments. **A**. FP competition experiments with menin variants bound to FLSN-MLL1$^{4-43}$ by MLL1$^{4-15}$. **B**. FP competition experiments with menin variants bound to FLSN-MLL1$^{4-43}$ by MLL1$^{4-28}$. **C**. Time dependent FP competition experiments with menin^{G331D} bound to FLSN-MLL1$^{4-43}$ by MLL1$^{4-15}$ and MLL1$^{4-28}$. Competition with menin^{WT} is included for comparison. Table shows maximal % inhibition at corresponding times. **D**. Comparison of the raw fluorescence signal between 3 and 72 h incubations from the FP competition experiments for the G331D variant.

Supplementary Tables

Supplementary Table 1. Analysis of thermodynamic parameters from ITC experiments for titrations of menin mutants with MLL1 fragments. A. Characterization of the binding by menin variants titrated with $MLL1^{160}$. Experiments were run twice. Errors were calculated by taking the standard deviation of all collected values for each type of experiment. T = 298.15 K for all experiments. **B.** Characterization of the binding by menin variants titrated with $MLL1^{4-15}$. Experiments were run twice apart from $menin^{G331D}$ and $MLL1^{4-15}$, which was run four times. Errors were calculated by taking the standard deviation of all collected values for each type of experiment. T = 298.15 K for all experiments.

A

Thermodynamic Parameter	WT	M327I	T349M	G331R	G331D
K_d (nM)	0.5 ± 0.3	2.2 ± 0.6	2.6 ± 0.7	1.3 ± 0.3	1.4 ± 0.7
Stoichiometry (N)	1.00 ± 0.05	1.04 ± 0.03	1.07 ± 0.06	1.14 ± 0.05	1.15 ± 0.09
ΔH (cal/mol)	-12570 ± 770	-12460 ± 210	-13450 ± 2200	-9100 ± 370	-13650 ± 1610
-TΔS (cal/mol)	-220 ± 1200	650 ± 370	1840 ± 2040	-3030 ± 220	1510 ± 1930

B

Thermodynamic Parameter	WT	M327I	T349M	G331R	G331D
K_d (nM)	48.9 ± 2.8	49.6 ± 5.4	49.4 ± 3.5	100.3 ± 10.9	524.5 ± 106.3
Stoichiometry (N)	1.32 ± 0.05	1.09 ± 0.04	1.31 ± 0.01	1.32 ± 0.06	1.24 ± 0.09
ΔH (cal/mol)	-10020 ± 350	-13220 ± 570	-9350 ± 1750	-7940 ± 1760	-9420 ± 1300
-TΔS (cal/mol)	50 ± 390	3250 ± 500	-620 ± 1830	-1610 ± 1830	850 ± 1240

Supplementary Table 2. Data Collection and Refinement Statistics for the menin mutants

**Data collection**	**Menin G331D**	**Menin G331R**	**Menin T349M**	**Menin M327I**
Space group	$P2_12_12_1$	$P2_12_12_1$	$P2_12_12_1$	$P2_12_12_1$
Cell dimensions				
a, *b*, *c* (Å)	48.73, 79.94, 124.71	48.71, 80.01, 124.62	49.02, 80.27, 124.94	49.00, 80.20, 124.65
α, β, γ (°)	90.0, 90.0, 90.0	90.0, 90.0, 90.0	90.0, 90.0, 90.0	90.0, 90.0, 90.0
Resolution (Å)	50-1.40 (1.42-1.40)	67.33-1.40 (1.47-1.40)	50-1.31 (1.33-1.31)	50-1.58 (1.61-1.58)
R_{sym} or R_{merge}	0.081 (0.852)	0.056 (0.573)	0.098 (0.707)	0.120 (0.823)
$I / \sigma I$	34.00 (2.13)	16.7 (2.5)	30.02 (2.31)	23.49 (2.03)
Completeness (%)	99.8 (100)	99.5 (98.6)	99.8 (98.3)	98.9 (97.0)
Redundancy	7.2 (7.1)	7.9 (8.0)	7.2 (6.1)	6.9 (6.1)
**Refinement**				
Resolution (Å)	24.96-1.40	67.33-1.40	28.92-1.31	31.18-1.58
No. reflections	91,574	91,457	112,984	64,300
R_{work} / R_{free}	0.165 / 0.190	0.158 / 0.181	0.162 / 0.183	0.161 / 0.194
No. atoms				
Protein	3771	3874	3740	3729
Ligand/ion	47	47	44	52
Water	506	509	590	450
B-factors ($Å^2$)				
Protein	18.1	17.6	13.7	18.9
Ligand/ion	39.4	39.6	34.2	43.2
Water	30.7	31.6	27.1	30.1
R.m.s. deviations				
Bond lengths (Å)	0.012	0.011	0.013	0.010
Bond angles (°)	1.993	1.985	2.024	1.816

All diffraction data were obtained from single crystals. Values in parentheses are for highest-resolution shell. Crystal structures were deposited in Protein Data Bank (PDB) under accession codes 9C4Z, 9C4W, 9C4Y and 9C4X, respectively.

Supplementary Table 3. Data Collection and Refinement Statistics for the menin mutant complexes with MLL1

Data collection	Menin G331D	Menin G331R	Menin T349M	Menin M327I
Space group	$P2_12_12_1$	$P2_12_12_1$	$P2_12_12_1$	$P2_12_12_1$
Cell dimensions				
a, *b*, *c* (Å)	49.08, 80.22, 124.93	48.70, 80.01, 124.36	49.05, 80.24, 125.09	49.08, 80.46, 124.95
α, β, γ (°)	90.0, 90.0, 90.0	90.0, 90.0, 90.0	90.0, 90.0, 90.0	90.0, 90.0, 90.0
Resolution (Å)	50-1.47 (1.50-1.47)	67.29-1.54 (1.62-1.54)	50-1.57 (1.60-1.57)	50-1.46 (1.49-1.46)
R_{sym} or R_{merge}	0.103 (0.923)	0.056 (0.569)	0.150 (0.963)	0.082 (0.726)
$I / \sigma I$	33.08 (2.09)	16.3 (2.5)	20.95 (2.03)	28.13 (2.27)
Completeness (%)	99.9 (100)	100 (100)	100.0 (99.9)	99.5 (97.1)
Redundancy	7.3 (7.2)	6.8 (6.8)	7.0 (5.6)	7.0 (6.6)
**Refinement**				
Resolution (Å)	27.83-1.47	67.29-1.54	29.15-1.57	31.13-1.46
No. reflections	80,456	68,925	66,223	81,677
R_{work} / R_{free}	0.160 / 0.188	0.172 / 0.202	0.160 / 0.197	0.156 / 0.184
No. atoms				
Protein	3753	3747	3806	3845
Ligand/ion	120	141	118	114
Water	531	397	503	533
B-factors ($Å^2$)				
Protein	19.7	22.5	17.5	16.5
Ligand/ion	34.9	33.0	30.5	28.0
Water	32.7	34.2	30.1	30.1
R.m.s. deviations				
Bond lengths (Å)	0.012	0.010	0.010	0.012
Bond angles (°)	1.914	1.905	1.852	1.995

All diffraction data were obtained from single crystals. Values in parentheses are for highest-resolution shell. Crystal structures were deposited in Protein Data Bank (PDB) under accession codes 9C4V, 9C4S, 9C4U and 9C4T, respectively.
